# Cranioplasty in Oman: Retrospective review of cases from the National Craniofacial Center 2012–2022

**DOI:** 10.18295/squmj.3.2024.017

**Published:** 2024-05-27

**Authors:** Khalifa Al Alawi, Asma Al Furqani, Sultan Al Shaqsi, Moath Shummo, Ahmed Al Jabri, Taimoor Al Balushi

**Affiliations:** 1 Department of Plastic & Reconstructive Surgery, Khoula Hospital, Muscat, Oman; 2 Department of Plastic & Reconstructive Surgery, University of Toronto, Toronto, Canada

**Keywords:** Bone Grafting, PEEK, Oman

## Abstract

**Objectives:**

Cranioplasty is a complex craniofacial and neurosurgical procedure that aims to reinstate the architecture of the cranial vault and elevate both its aesthetic and neurological function. Several reconstructive materials have been thoroughly explored in the search for the optimal solution for cranioplasty. This study aimed to evaluate different material used for cranial reconstruction in Oman.

**Methods:**

This retrospective study included all patients who had had cranioplasty procedures performed at Khoula Hospital, Muscat, Oman, from 2012 to 2022. Demographic information, the characteristics of the cranial defect and any complications that occurred post-operatively were analysed.

**Results:**

A total of 47 patients were included in this study. The most common cause of cranial defects was craniectomy following traumatic head injury (70.2%) along with excision of fibrous dysplasia (10.6%). The most frequently utilised material for cranial repair was autologous bone grafts (n = 28), followed by polyetheretherketone (PEEK; n = 14). Interestingly, the replacement of bone grafts from previous craniectomy showed a notably high resorption rate (71.4%), in contrast to split calvarial grafts (0%) and other types of bone grafts (14.3%). Additionally, delayed graft infection was observed in 3.6% of the bone graft group and 7.1% of the PEEK group.

**Conclusion:**

Patient-specific alloplastic implants such as PEEK have gained popularity for large and complex cranioplasty, as they provide excellent aesthetic outcomes and leave no donor site morbidity. In contrast, bone grafts remain the gold standard for small to medium-sized cranial defects.

**Advances in Knowledge**

- *This study provides a detailed description of the causes of cranial defects in Oman. Considering road traffic accidents (RTAs) are one of the leading causes of traumatic cranial defects, it is important to highlight the importance of RTA safety precautions.*
- *This study emphasises case-to-case-based material selection.*

**Application to Patient Care**

- *This study provides a better understanding of the causes of cranial defects in Oman, which helps in designing better prevention strategies and preparing the healthcare system in Oman.*
- *It provides a guide for craniofacial plastic surgeons on the material selection for cranial reconstruction.*
- *The study emphasises the importance of strict preservation conditions for autologous bone grafts.*
- *It highlights the importance of strict infection prevention protocols during cranial reconstruction surgeries.*

The human skull is a unique bony structure that plays an essential role in the distinctive appearance of an individual.^1^ It also acts as a protective vault for the central nervous system. However, this sophisticated structure can be disrupted by multiple disease processes, such as trauma and malignancies, which lead to cranial defects. Defects in the skull can be caused by trauma, decompressive craniectomies, congenital anomalies or tumour resections.^2^ This loss of bone compromises the skull’s function as a brain guard and leaves the brain vulnerable to further physical trauma.^3^ In addition, the absence of a sizeable calvarial bone results in several physiological and psychological complications.^1^–^4^

The skull shape contributes significantly to physical appearance (i.e. any defect in this area will result in extreme disfigurement). Pruzinsky illustrated that individuals with major craniofacial abnormalities might experience social withdrawal and develop psychological and emotional distress.^5^ Among the other complications of absent cranial bony coverage is the ‘syndrome of trephine’, described in 1939 by Grant and Norcross.^6^ Patients experience a cluster of symptoms, including headache, insomnia, behavioural changes, vertigo, tinnitus and fatigue.^4^^,^^7^ The ‘sinking scalp flap syndrome’ has also been used to describe focal motor deficits in patients who undergo craniectomy and have a persistent hemi-cranial defect. It is also known as motor trephine syndrome.^8^ Due to the many complications of cranial defects, cranial reconstruction is performed.^1^^,^^9^

The main goal of cranioplasty is to restore the function of the skull shield, provide symptom relief and enhance the patient’s aesthetics.^1^^,^^3^^,^^9^^,^^10^ A study involving 62 patients demonstrated that cranioplasty significantly improved the quality of life in all aspects during a 24-month follow-up.^11^ This improvement was measured using short form-36, an assessment tool consisting of 8 main domains (physical functioning, role physical, bodily pain, general health, vitality, social functioning, role emotional and mental health).^11^^,^^12^ Moreover, numerous studies have observed that cranioplasty enhances cerebral blood flow (CBF) in both hemispheres.^13^–^17^ This increase in CBF appears to be a significant contributor to the symptomatic relief experienced by patients after reconstruction, including the resolution of headaches. Another study, utilising objective measurement tools such as Glasgow Outcome Scale, Frontal Assessment Battery and Mini Mental State Examination, demonstrated cognitive recovery in 92% of the participants during a 6-month follow-up.^13^ Consequently, it was concluded that cranioplasty plays a vital role in the neurological and psychosocial rehabilitation of patients with skull defects.^10^^,^^17^^,^^18^

Several reconstructive materials have been developed and used to close cranial defects. These materials are broadly categorised into 2 main groups, biological and synthetic. Biological materials include autologous grafts, allografts and xenografts.^1^^,^^7^^,^^19^ The first documented use of a bone xenograft dates back to 1668 when van Meekeren reported the use of canine bone for the reconstruction of the skull of a Russian noble.^9^ Later, Walther conducted the first successful case of autologous bone grafting in 1821.^6^^,^^19^ However, xenografts were greatly discouraged later on due to their high infection, rejection and resorption rates.^20^ It was not until the early 20^th^ century that the use of autografts became widely practised for their advantages, such as high biogenic compatibility (resulting in a low rejection rate) and their moulding and integration ability into bones, especially in the paediatric age group where bones are still growing.^19^^,^^21^ Several synthetic materials have been used, starting with acrylic after World War II. Subsequently, many other materials were developed and employed. However, due to side effects, technical difficulties and limited accessibility associated with them, these materials are not utilised in current practice.^22^–^24^ Nonetheless, materials such as polyetheretherketone (PEEK), titanium mesh and alumina ceramics are widely employed in contemporary practice, demonstrating favourable outcomes, including a low infection rate, shorter operation time, low resorption rate and enhanced strength.^25^^,^^26^

The complication rates among cranial reconstructive materials differs. For instance, both bone xenografts and allografts have a high rate of infection and resorption.^6^^,^^20^ Bone autografts are the gold standard for closing small and medium cranial defects after the decompressive procedure because of their low infection rate and cost and ready availability. However, they carry a high risk of bone resorption and breakdown, especially in children.^19^^,^^27^^,^^28^ Synthetic materials show a lower infection rate, resorption and need for revision surgery, along with favourable cosmetic outcomes due to constant advancements in computer-based customisation and 3-dimensional (3D) printing.^21^^,^^25^^,^^26^ Furthermore, among different synthetic materials, titanium mesh has the lowest infection rate and a higher cosmetic outcome. However, it is also found to be heat-conductive and considered more costly.^19^^,^^25^^,^^29^ Methyl methacrylate is strong, radiolucent and non-conductive to heat, but is unfortunately associated with a high infection rate.^19^^,^^25^^,^^30^^,^^31^ The use of hydroxyapatite is limited because of its high infection rate, limited osteointegration and low tensile strength, leading to fragmentation, although its flexibility and expansion properties make it favourable for use in the paediatric age group.^19^^,^^32^ Alumina ceramics and PEEK have the desired strength, low infection rate and a favourable cosmetic outcome and are chemically stable, but they are considered the most expensive materials and lack osteogenic properties.^19^^,^^23^^,^^25^^,^^33^

In Oman, the vast majority of cranioplasties are performed at the national trauma centre, Khoula Hospital, Muscat, Oman. Cases of cranial reconstruction involving the replacement of bone from previous craniectomy procedures are exclusively handled by neurosurgeons. In contrast, instances of cranioplasty using other types of bone grafts or allograft materials, as well as those involving complex cranial defects, are mainly undertaken by the craniofacial plastic team. While a study has been published on Oman’s experience with PEEK cranioplasty, there are no reports of cranial reconstruction using other materials.^34^ Hence, this study was conducted to fill that gap.

## Methods

This retrospective cross-sectional study was conducted between February 2012 and December 2022 and included all cases of cranial reconstruction performed in Khoula Hospital. The initial participant list was retrieved from the medical electronic files of Khoula Hospital (Al Shifa 3 Plus) using keywords such as cranioplasty, cranial reconstruction, PEEK, titanium and bone graft. After a thorough review of the initial list was conducted, only patients who satisfied the inclusion criteria were included; patients who underwent immediate cranioplasty post-craniotomy and cases of reduction of cranial bone fractures were excluded.

The electronic medical records of these patients were extensively examined to extract study parameters. Demographic information, such as age and gender, was collected. Details regarding cranial defects, including the mechanism, location, size and any prior reconstruction, were recorded as well as cranial reconstruction parameters such as the type of material, operative time and hospital stay. Additionally, immediate and delayed postoperative complications were identified. Delayed adverse outcomes were defined as complications that occurred after the patient was discharged postoperatively. The screened complications included wound infection, seizure, hydrocephalus, haematoma, significant seroma requiring aspiration, subdural hygroma, wound gaping, bone resorption, implant exposure, hardware failure and revision surgery. To mitigate bias, data were independently collected by two trained researchers. All data were coded and stored in a password-protected computer, consolidated in a single Excel sheet (Microsoft, Redmond, Washington, USA).

This study was approved by the Khoula Hospital Ethical Board (PRO052022120), and conducted per the Declaration of Helsinki. Patient consent was obtained, and patients signed a written consent to share their data and have their photos used in this publication.

## Results

A total of 47 cases were included in this study. These patients had cranial defects and underwent cranioplasty that involved various cranial reconstruction materials. Most cases had no known medical comorbidities, except for 3 patients with hypertension, 3 patients who had multi-suture craniosynostosis and had been previously operated on and 1 diabetic patient.

The most common cause of cranial defects was trauma, accounting for 33 cases (70.2%), with 26 defects developing post-road traffic accidents (RTAs), 6 cases occurring post-falls and 1 case caused by a gunshot injury. A total of 5 cases had cranial defects after fibrous dysplasia excision (10.6%). Other causes of cranial defects in this series included excision of Langerhans cell histiocytosis (4.3%), squamous cell carcinoma (4.3%), neurofibroma (2.1%), frontal encephalocele (2.1%), cleidocranial dystocia (2.1%), post-debridement of osteomyelitis area (4.3%) and decompression craniotomy for brain abscess drainage (2.1%).

A prior history of cranial reconstruction was recorded for 13 patients. Among these, 7 patients had reconstruction using bone autografts, 1 with titanium mesh and 5 with other methods, including elevation of the fractured segment and fixation, fronto-orbital advancement and cranial vault expansion. Most cranial reconstructions in these cases were done using bone autografts (n = 28, 59.6%). In the bone graft category, 14 cases were reconstructed using split-thickness calvarial bone, 7 cases using bone from previous craniectomy (known as bone replacement), and another 7 cases using bone grafts from other locations such as the iliac crest and rib [Figure 2]. PEEK was used in 14 cases (29.8%), 2 cases were reconstructed with bone cement (4.3%), 2 cases with titanium mesh (4.3%) and 1 case with acrylic (2.1%) [Figure 3].

In terms of the locations of these cranial defects in the study sample, 11 were fronto-temporo-parietal (23.4%) in location, 10 were frontal defects (21.3%), 6 were fronto-parietal defects (12.8%) and 4 were defects in the fronto-temporal area (8.5%). There were 5 cases involving parietal defects (10.6%) and the following other defects were seen: occipital (6.4%), temporal (2.1%), parieto-occipital (8.5%) and temporo-parietal (4.3%). The mean size of the defects was 80.6 cm^2^. The largest defect among the study cases was 300 cm^2^, whereas the smallest was 5.25 cm^2^.

There were no intra-operative complications and the mean operation time was 3 hours and 56 minutes. Furthermore, the researchers investigated the average operation time for each used material and found that the longest average time for performing cranial reconstruction was using bone autografts (3 hours and 57 minutes). The average hospital stay was 10.8 days [Table 1].

**Figure 1:**
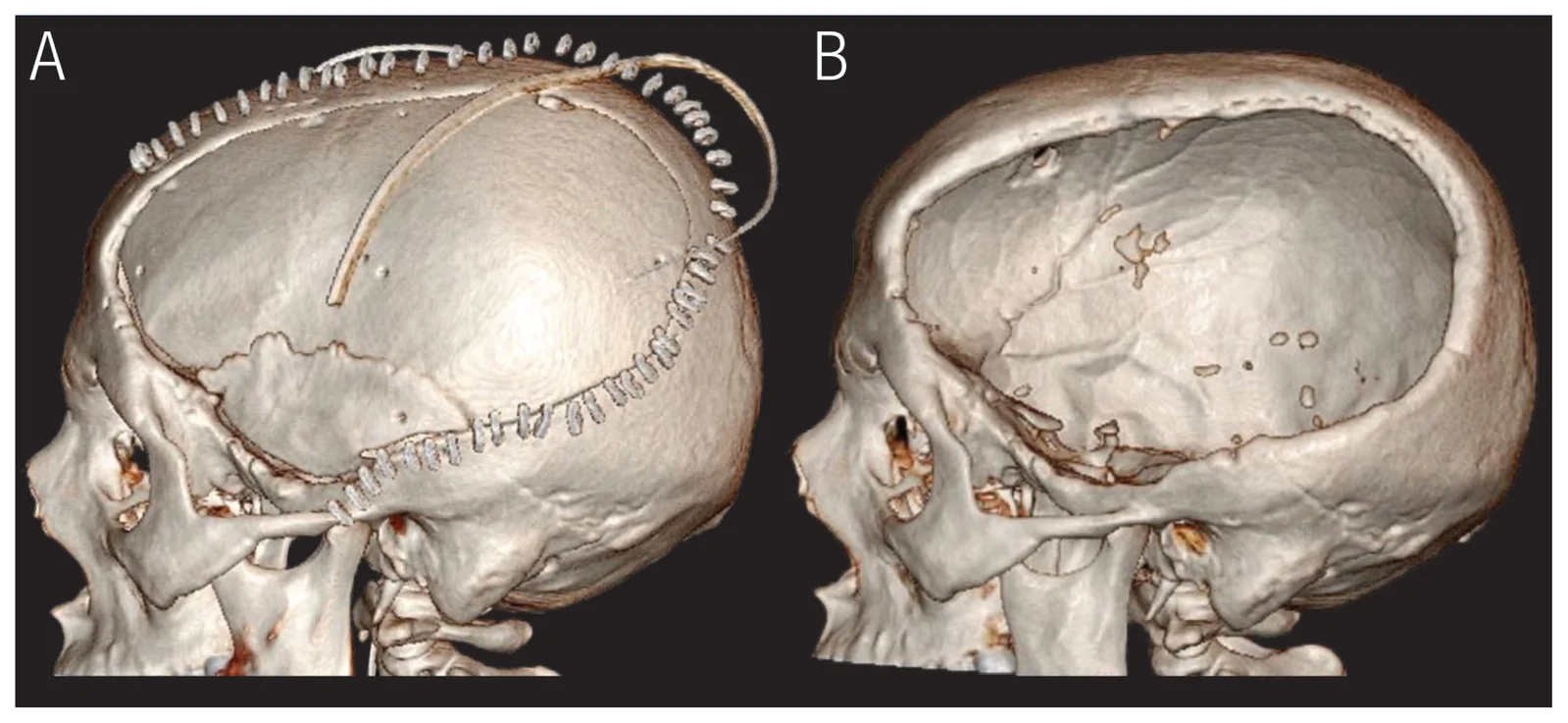
A computed tomography scan with 3D reconstruction of the right side of the skull **(A)** third-day post cranioplasty with bone graft replacement (bone from the previous craniectomy) and **(B)** 9 months post-operative showing severe resorption of the bone graft (>98).

**Figure 2:**
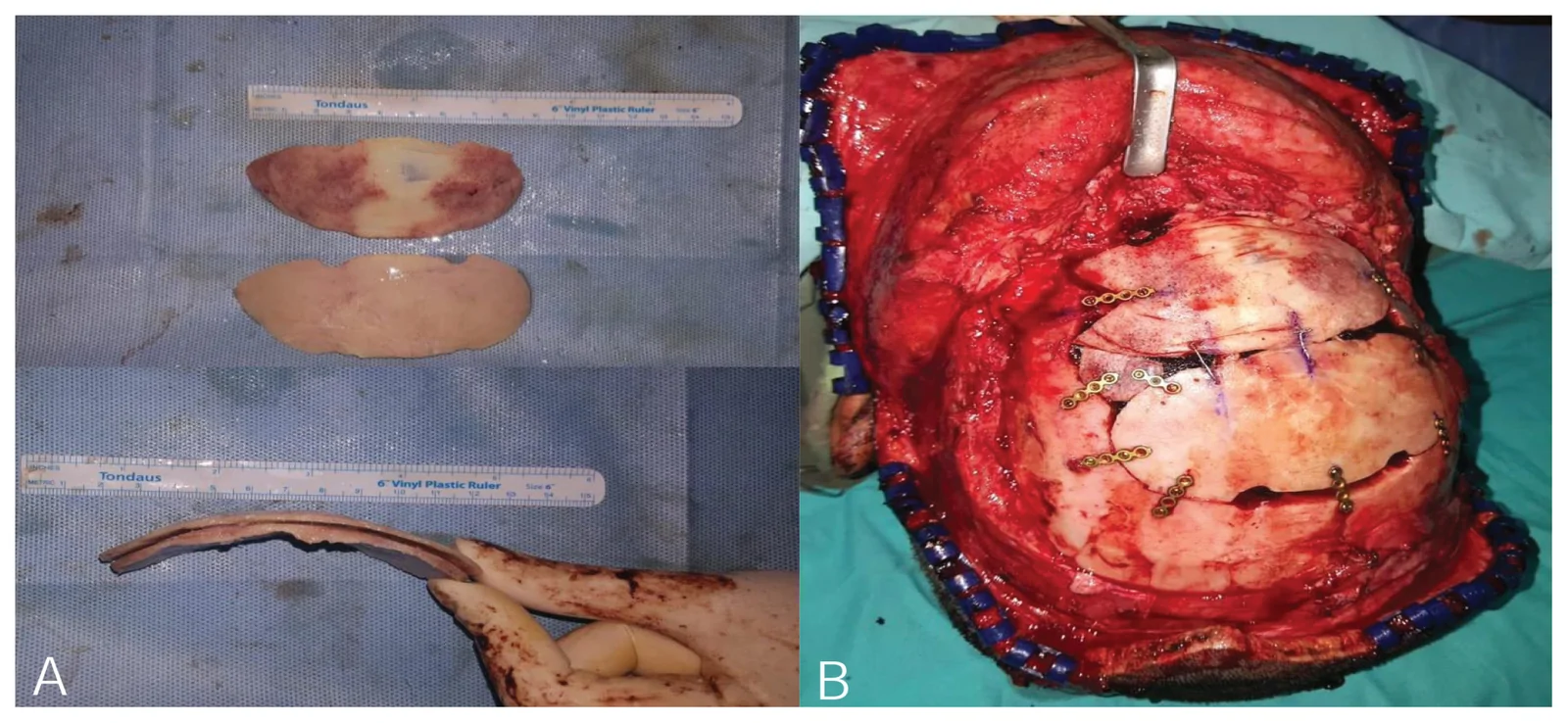
Photographs showing **(A)** the splitting of the calvarial bone in the anterior and posterior and **(B)** postresection of fibrodysplasia cranial reconstruction with split-thickness calvarial bone graft.

**Figure 3:**
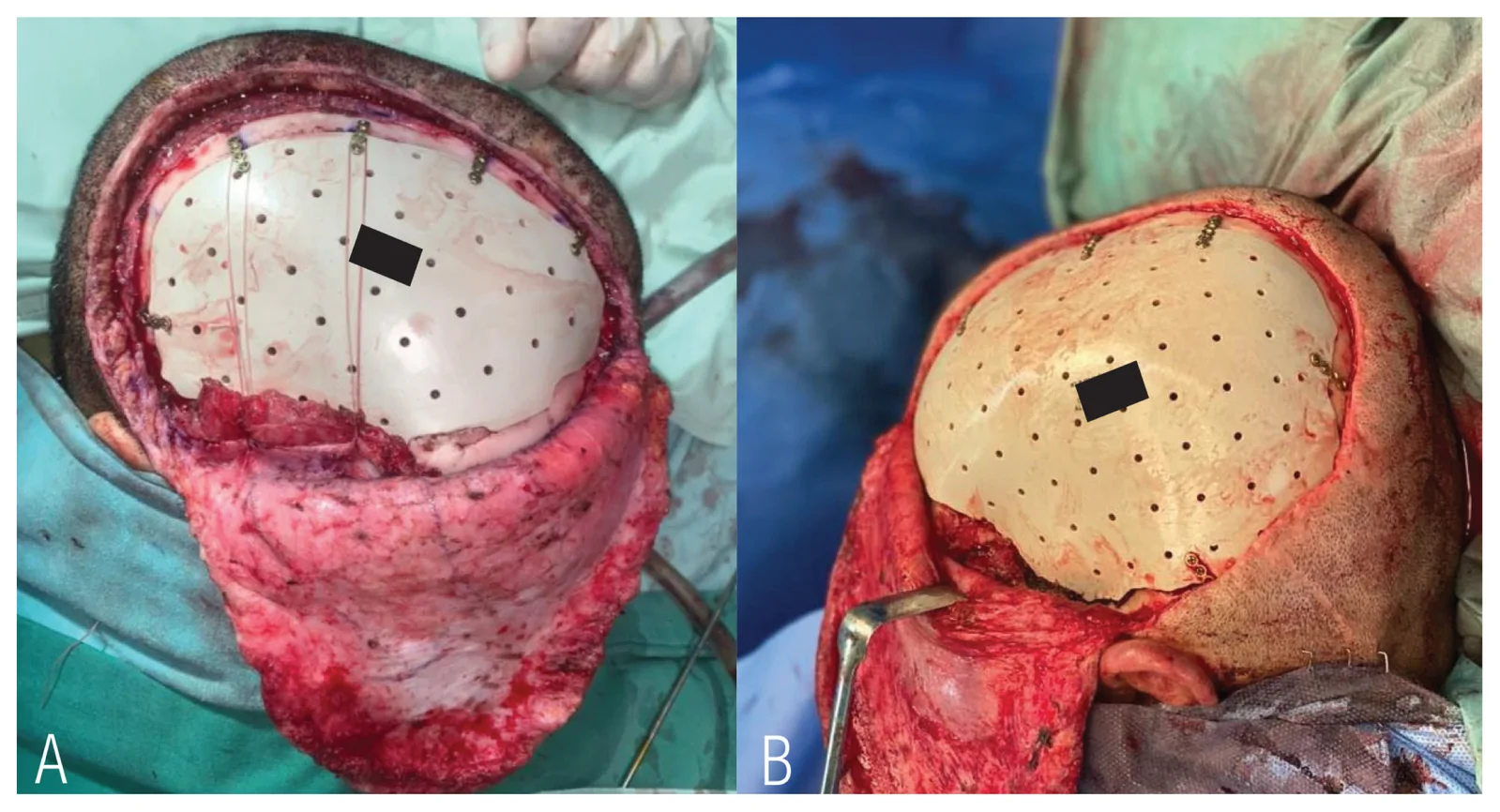
Photographs showing **(A)** the temporalis muscle being suspended over a polyetheretherketone (PEEK) patient-specific cranial implant and **(B)** PEEK cranial implant without temporalis muscle resuspension.

Immediate post-operative complications were observed in 4 cases (8.5%); 3 cases developed a haematoma, and one had a wound infection. Delayed complications developed in 40.4% of the cases, with some cases experiencing multiple adverse outcomes. The most frequent delayed complications were significant bone graft resorption (n = 6) and residual deformity (n = 5). Additionally, delayed graft infection was observed in 3.6% of the bone graft group and 7.1% of the PEEK group. The majority of bone graft loss occurred in cases where bone from a previous craniectomy was used (71.4%) [Table 2 and Figure 1].

## Discussion

In the current study, 47 patients with cranial defects underwent cranioplasty between 2012 and 2022. The most common cause of cranial defects was traumatic, predominantly post-RTAs. In 2012, RTAs were reported to be the top cause of injuries, disabilities and deaths in Oman, according to an official report by the Omani Ministry of Health. In a subsequent analytic study, the rate of RTAs was observed to have a minimal decline until 2018.^35^^,^^36^ Piitulainen *et al*. conducted a retrospective observational study to assess the operative outcomes of cranioplasty after severe traumatic brain injury treated with decompressive craniectomy.^37^ They reported that a successful cranioplasty predicted favourable patient outcomes one year after the procedure. Moreover, it was reported that the appearance of traumatic subarachnoid haemorrhage on imaging was a major risk factor for implant removal.^38^

As the ideal material for cranial defect reconstruction remains a matter of debate, the current study utilised different materials, with decisions made based on various factors, including but not limited to the size and location of the defect, availability of materials and surgeon preference. Overall, bone autografts were the most frequently used material, accounting for 59.6% of cases. Most of these patients underwent calvarial split-thickness bone grafting and 78.5% of them had small-to-medium–sized defects (<100 cm^2^). Among the 28 patients who underwent bone autografting for cranial defects, 6 experienced bone graft resorption (21.4%) and graft migration was observed in 2 cases. Cranial reconstruction with bone graft stored from the previous craniectomy, also known as bone replacement, exhibited the highest resorption rate compared to other types of bone autograft, with a percentage of 71.4%. A single case with a prior history of regional radiotherapy underwent repair using a rib bone graft and this was complicated by multiple infections and bone resorption, ultimately leading to graft removal. However, all other bone autograft sources showed no resorption after a 1-year follow-up. In a meta-analysis published in 2016, the resorption rate was found to be 9.7% after decompressive craniotomy, with an average storage duration of 69.9 days and a mean freezing temperature of −57°C.^39^ In addition, bone graft resorption can still occur beyond 12 months postoperatively. For example, a randomised controlled trial that followed-up with 31 patients who received titanium cranial implants and 31 patients who underwent autologous bone cranioplasty in the previous 24 months showed bone resorption during long-term follow-up.^40^ Cabbad *et al*. concluded that autologous bone was still the most reliable, safe and cost-effective material for cranioplasty.^41^ It remains the gold standard due to its excellent biocompatibility and osteogenesis ability. However, its use is hindered by its tendency for resorption and the need for preservation. It is usually either preserved at freezing temperatures (−70°C) or within the abdominal wall.^42^ When comparing the 2 methods of preservation, Corliss *et al*. found no statistically significant differences in terms of infection, resorption and reoperation rates.^39^ However, most centres nowadays avoid opening the abdominal wall for preservation to minimise additional surgery scarring and comorbidities.^1^ In the current study centre, the current neurosurgical practice is to preserve the bone graft from craniectomy in a freezer at −5°C. In addition, a recently published study in 2023 suggested a new way of preserving bone grafts in the freezer to reduce infection later. Their novel cryopreservation approach involved placing the bone graft in gauze saturated with 80 mg of gentamicin and 2 g of nafcillin within a 3-layer sterile bag system. They managed to reduce the infection rate from 18.7% using the traditional wet cryopreservation method to 5.6% using the new dry cryopreservation method.^42^ Furthermore, the majority of these cases involve complicated motor vehicle collision victims who underwent cranioplasty late, during which the bone remained in place for an extended period, exceeding a year in most instances. This delay in reconstruction and suboptimal preservation might explain the high rate of resorption observed in cranioplasty with bone replacement. In a study conducted in South Korea investigating the risk factors for bone resorption, it was concluded that the paediatric age group, larger skull defect, the gap between the bone flap and bone edge and heat sterilisation of autologous bone could be contributing factors for bone resorption.^43^ Additionally, a multicentre study reported that it would take two years to stabilise the bone flap and therefore recommended a 2-year follow-up as an optimal time frame.^44^

Custom-made PEEK implants exhibit superior aesthetic outcomes as it is patient-specific. An analysis of 12 patients with PEEK implants using root mean square error between the presurgical virtual position and the postoperative actual position of the implant revealed that PEEK implants manufactured in a patient-specific style demonstrated highly accurate positioning. This, in turn, resulted in superior aesthetic outcomes.^45^ Besides its cost, PEEK implants lack osteogenic properties and the ability to integrate with surrounding bones, which might increase the risk of infection, local inflammation and dislodgment.^19^^,^^21^^,^^26^ Patient-specific PEEK implants were used in 14 cases (29.8%), with no intra-operative or immediate complications. However, seroma was noted in 4 cases (28.6%), implant migration in 1 case (7.1%) and seizures in 2 (14.3%). In comparison to the results of Punchak *et al*.’s meta-analysis, the incidence in the current study tends to fall within the international range.^46^ The incidence of infection post-cranioplasty ranges from 5–33% worldwide and the study rate was 6.4% across all used materials and 7.1% with PEEK implants.^47^–^51^ The case of the infected PEEK implant involved a 44-year-old male with fibrous dysplasia. This patient underwent left frontal bone and superior orbital resection, along with frontal sinuses obliteration. Simultaneously, 2 PEEK patient-specific implants were used for reconstruction. The patient initially recovered well without complications. However, on a 4-year follow-up, the patient developed intermittent clear nasal discharge. Computed tomography (CT) scans and other laboratory tests were conducted. Cerebrospinal fluid rhinorrhea was ruled out and no clinical or laboratory findings suggested infection. The patient was continued on conservative management. The low infection rate in the study could be explained by the strict protocol, which was defined in a previous study; it consists of intravenous cefazolin for a total of 5 days, in addition to frequent and extensive head washing with chlorhexidine preoperatively.^34^

Regarding the size, the largest defect among the cases studied was 300 cm^2^, whereas the smallest was 5.25 cm^2^. The mean operative time for all cases in the study was 3 hours and 56 minutes. Multiple studies, including Sedney *et al*.’s study, have argued that a larger craniectomy size improves survival without the risk of increased complications.^52^ On the other hand, larger defects may require more meticulous surgical techniques, leading to longer operative times that may increase the risk of surgical site infection; Shibahashi *et al*. stated that the estimated 2-year surgical site infection risk was 31.3% for the long operative time (>1 hour and 38 minutes).^53^

In the current study’s current craniofacial protocol, the researchers advise against using preserved bone grafts from previous craniectomy to reconstruct large cranial defects, especially if the graft was not stored in an optimal environment, as it carries a high resorption rate. Alternatively, it is recommended to use patient-specific PEEK implants for large defect cranioplasty, as it has superior aesthetic outcomes and excellent survival. In contrast, a split calvarial bone graft is an optimal option for the reconstruction of small to medium defects.

In terms of follow-up, the current study protocol involves close interval monitoring during the first 6 months postoperative and subsequently every year. According to some studies, the standard follow-up is recommended at 3 months.^54^ However, certain complications can still arise in the long term, such as bone resorption or delayed implant infection.^40^ Therefore, the researchers adhere to a rigorous long-term follow-up, extending up to 5 years in some cases. Concerning imaging investigations, CT maxillofacial with 3D reconstruction is performed on the third day postoperative, followed by additional assessments at 3 and 6 months postoperative. Moreover, delayed CT scans can be conducted to evaluate for late complications such as assessing the extent of bone resorption beyond the 12-month follow-up period.

## Conclusion

Cranial reconstruction remains a matter that requires more research given the wide variety of available materials and their variable success and complication rates. Thus, material selection should be tailored based on the defect characteristics. Additionally, there is a need to develop more optimal materials that offer good biocompatibility, infection resistance, a high survival rate and provide a satisfying aesthetic outcome.

## Figures and Tables

**Table 1:** Mean operation time and mean hospital stay among each used material.

	Mean operation time	Mean hospital stay in days
**Bone autograft**		
Calvarial split BG	4 hours 47 minutes	7
BG from craniotomy	2 hours 10 minutes	25
Rib BG	4 hours 50 minutes	8
Iliac crest BG	2 hours 40 minutes	14
**PEEK**	3 hours 51 minutes	8
**Cement**	5 hours	7
**Titanium**	2 hours	Not known
**Acrylic**	2 hours	3

BG = bone graft; PEEK = polyetheretherketone.

**Table 2:** Materials used and observed complications in patients who underwent cranioplasty

Material	Complication, n (%)							
	Immediate		Late					
	Wound infection	Haematoma	Bone resorption	Delayed graft infection	Graft migration	Residual deformity	Seroma	Seizure
**Bone autograft**								
Calvarial split BG (n = 14)	0 (0.0)	2 (14.3)	0 (0.0)	0 (0.0)	0 (0.0)	1 (7.1)	0 (0.0)	0 (0.0)
BG from craniectomy (n = 7)	1 (14.3)	0 (0.0)	5 (71.4)	1 (14.3)	1 (14.3)	0 (0.0)	1 (14.3)	0 (0.0)
Other distant BG (n = 7)	0 (0.0)	0 (0.0)	1 (14.3)	0 (0.0)	1 (14.3)	0 (0.0)	0 (0.0)	0 (0.0)
**PEEK** (n = 14)	0 (0.0)	0 (0.0)	0 (0.0)	1 (7.1)	1 (7.1)	2 (14.3)	4 (28.6)	2 (14.3)
**Titanium mesh** (n = 2)	0 (0.0)	0 (0.0)	0 (0.0)	1 (50.0)	1 (50.0)	0 (0.0)	0 (0.0)	0 (0.0)
**Acrylic** (n = 1)	0 (0.0)	0 (0.0)	0 (0.0)	0 (0.0)	1 (100.0)	0 (0.0)	0 (0.0)	0 (0.0)
**Bone cement** (n = 2)	0 (0.0)	0 (0.0)	0 (0.0)	0 (0.0)	0 (0.0)	1 (50.0)	0 (0.0)	0 (0.0)

BG = bone graft; PEEK = polyetheretherketone.
